# Superimmunity by pan-sarbecovirus nanobodies

**DOI:** 10.1016/j.celrep.2025.116023

**Published:** 2025-07-01

**Authors:** Yufei Xiang, Wei Huang, Hejun Liu, Zhe Sang, Sham Nambulli, Jérome Tubiana, Kevin L. Williams, W. Paul Duprex, Dina Schneidman-Duhovny, Ian A. Wilson, Derek J. Taylor, Yi Shi

## Main text

(Cell Reports *39*, 111004; June 28, 2022)

As this paper was originally published on June 23, 2022, it mistakenly included the NIH grant RM1GM142002 as a funding source. The authors apologize for the error.

